# Emergency management: optic nerve compression

**Published:** 2018-11-09

**Authors:** Colin Cook

**Affiliations:** 1 Professor and head of the Division of Ophthalmology: University of Cape Town, Groote Schuur Hospital, Cape Town South Africa.

**Increased orbital pressure compresses the optic nerve, which can lead to irreversible vision loss in a matter of hours.**

**Figure 1:**
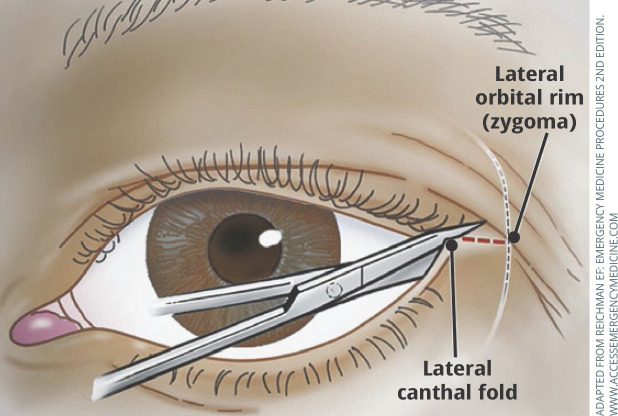
Performing a surgical canthotomy will relieve pressure on the optic nerve. This can save the person's sight if done in time.

The orbit is a bony box containing the eye and surrounding soft tissues, including part of the optic nerve. A significant increase in the pressure inside the orbit can reduce orbital blood supply and compress the optic nerve, causing damage. In acute cases, irreversible visual loss can occur after 90–120 minutes, unless the pressure is reduced and the blood supply restored.^1^

## What are the symptoms and signs of optic nerve compression?

In addition to a possible history of trauma, surgery or proptosis (forward displacement of the eye), the patient with optic nerve compression can present with the following:

- Pain
- Visual complaints (e.g., blurred or double vision and peripheral, slow, intermittent or sudden visual loss)
- Unexplained nausea and vomiting.

Clinical signs of optic nerve compression include:

- Decreased visual acuity
- Resistance to retropulsion of the eye (a ‘tense’ eye)
- Increased intraocular pressure
- Relative afferent pupillary defect^2^
- Inability to read colour vision plates (due to colour blindness).

## Causes of orbital compression

**Soft tissue swelling.** This can be due to tumours, trauma, infections or inflammation (e.g., orbital cellulitis or dysthyroid eye disease).

**Haematoma.** Blunt or penetrating injury to the orbit may result in haemorrhage in the orbit. The resulting haematoma can compress the optic nerve.

**Air in the orbit.** An orbital fracture can result in optic nerve compression. If a patient with an orbital fracture blows her or his nose, air may be forced from the paranasal sinus through the fracture and into the orbit, causing pressure on the optic nerve.

## Management

### Optic nerve compression due to soft tissue swelling or a haematoma

If you suspect optic nerve compression due to soft tissue swelling or a Haematoma, perform a lateral canthotomy to decompress the orbit (see panel).

Optic nerve compression due to soft tissue swelling may also be treated using systemic steroids. Ideally, this would be with 1 g intravenous methyl prednisolone daily for 3 days. However, if this drug is unavailable, oral prednisolone 1 mg/kg for 5 days would be a reasonable substitute. These are high doses of steroids, so it is important to watch for possible side-effects.

In acute cases, you may need to aspirate blood from a haematoma. Use a 5 ml syringe and the same needle placement that is used when administering a peribulbar local anaesthetic.

### Optic nerve compression due to air in the orbit

Patients with an orbital fracture should be instructed not to blow their nose for at least six weeks to allow the fracture to heal and to prevent optic nerve compression.

If a patient does blow their nose after an orbital fracture, and they experience a sudden loss of vision in the eye, with proptosis and an afferent pupil defect, they should immediately have the orbit decompressed. Release the air in the orbit using a 5 ml syringe and the same technique that is used to aspirate blood from a haematoma. This should result in an immediate improvement in optic nerve function and vision.

## Emergency kit

The contents of an emergency kit to deal with optic nerve compression should include:

- Local anaesthetic for a lateral canthotomy
- Straight scissors for a lateral canthotomy
- 5 ml syringe and 21-gauge needle to aspirate blood or air from the orbit.

**Performing a lateral canthotomy**

- If you think it needs to be done, do it as soon as possible
- Infiltrate the lateral canthus with local anaesthetic
- Use sharp, straight scissors. Make a good, clean cut about 1 cm in length (see Figure 1) from the lateral canthal fold to the lateral orbital rim (zygoma)
- Leave the wound open. Repair once the intraocular pressure has decreased.
